# When CAR Meets Stem Cells

**DOI:** 10.3390/ijms20081825

**Published:** 2019-04-12

**Authors:** Jung Min Lee

**Affiliations:** School of Life Science, Handong Global University, Pohang 37554, Korea; jm.lee@handong.edu; Tel.: +82-54-260-1292

**Keywords:** pluripotent stem cell, CAR, CRISPR/Cas9

## Abstract

The generation of immune cells from human pluripotent stem cells (embryonic stem cells and induced pluripotent stem cells) has been of keen interest to regenerative medicine. Pluripotent stem cell-derived immune cells such as natural killer cells, macrophages, and lymphoid cells, especially T cells, can be used in immune cell therapy to treat incurable cancers. Moreover, since the advent of chimeric antigen receptor (CAR) technology, the success of CAR-T cells in the clinic has galvanized new efforts to harness the power of CAR technology to generate CAR-engineered immune cells from pluripotent stem cells. This review provides a summary of pluripotent stem cell-derived immune cells and CAR technology, together with perspectives on combining pluripotent stem-cell derived immune cells and CAR engineering to pave a new way for developing next generation immune cell therapy.

## 1. Introduction

Human pluripotent stem cells—embryonic stem cells (ES cells) and induced pluripotent stem cells (iPS cells)—are cell types that can theoretically give rise to all of the cells of our body and proliferate indefinitely, providing an inexhaustible source for immune cell generation [1]. Differentiated immune cells can be utilized as an invaluable source for immune cell therapy to treat tumors that are incurable by conventional methods [2,3]. Chimeric antigen receptor (CAR) is an innovative technology that enables cells to navigate specifically to their target tumors [4]. With the remarkable complete response rates induced by CD19 CAR-T cells, two CAR-T cell therapies were approved by the U.S. FDA in 2017 [5]. After the success of the first CAR-T product on the market, a tremendous effort is being applied to develop more CAR-T cell therapies. To make immune cell therapy safer, more specific, and more cost-effective, gene editing and clonal selection is becoming recognized as essential. However, primary immune cells are refractory to gene editing procedures and such cells have limited proliferation activity, which hinders clonal selection. Therefore, with their capacity for indefinite proliferation and their pluripotent character, human pluripotent stem cells could be a perfect alternative for generating an unlimited number of improved immune cells through gene modification and clonal selection [6]. This review will summarize the derivation of immune cells [natural killer (NK) cells, macrophages, and T cells] from human pluripotent stem cells, and will provide perspectives on combining pluripotent stem-cell derived immune cells, gene editing, and CAR engineering to pave a new way for developing next generation immune cell therapy.

## 2. Pluripotent Stem-Cell Derived Immune Cells

**Figure 1:**
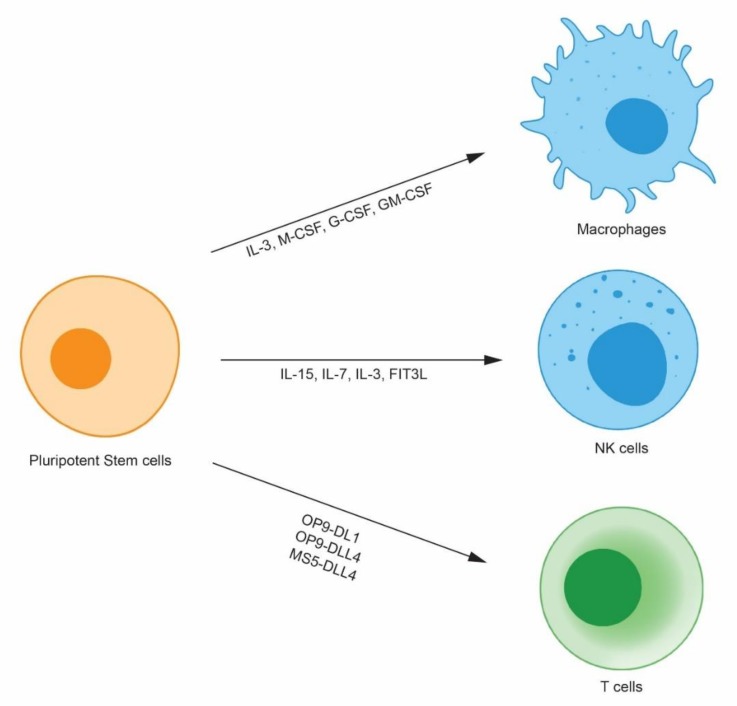
Human pluripotent stem cell-derived immune cells. Human pluripotent stem cells differentiate into immune cells such as macrophages, natural killer (NK) cells, and T cells by virtue of cytokines and stromal cells. Because human pluripotent stem cells proliferate indefinitely, they can be clonally selected after gene modification and can provide an unlimited number of modified immune cells.

In 1998, the first human ES cells were established by the Thomson group [7]. Human embryos were cultured until the blastocyst stage, 14 inner cell masses were isolated, and five ES cell lines were derived. Even though human ES cells hold great potential as therapeutic cells, ethical issues remain to be resolved due to the utilization of fertilized eggs. In 2006, a monumental discovery impacted the field of translational medicine. Dr. Yamanaka discovered that mouse somatic cells can be reprogrammed to ES cell-like cells by only four transcription factors; the cells were termed iPS cells [8]. Mouse iPS cells have demonstrated self-renewal and differentiation activity comparable to ES cells. Soon after, the success of iPS cell generation from mouse somatic cells was applied to generate human iPS cells. A year after the first mouse iPS publication, two papers reported human somatic cell reprogramming to iPS cells [9,10]. Because human iPS cells can be generated from fully differentiated somatic cells, even from cells in the urine [11], iPS cells are relatively free from the ethical issues faced by human ES cells. In addition, human iPS cells can be re-introduced into patients by autologous transplantation without inducing severe immune rejection. Therefore, there has been much interest in using iPS cells to generate therapeutic cells, such as hematopoietic stem cells [12], retinal pigment epithelial cells [13], and pancreatic beta cells [14]. Immune cells are invaluable cell types that can be derived from iPS cells for immune cell therapy. Such derived immune cells can be used to treat tumors that are not curable through conventional treatments in the clinic such as chemotherapy and radiation therapy. Here, the generation of three immune cell types, NK cells, macrophages, and T-cells, will be discussed (Figure 1). 

### 2.1. Pluripotent Stem Cell-Derived Natural Killer Cells

NK cells are critical players in the innate immune system that induce lysis of virus-infected cells and tumors. However, unlike cytotoxic T cells, they do not require immunization [15,16]. NK cells utilize their receptors to respond to specific molecules expressed on virus-infected cells and tumor cells. The NK cell activating-receptors such as Ly49H [17], NKp46 [18], and NKG2D [19], respectively, recognize murine cytomegalovirus infection, hemagglutinin from influenza virus/hemagglutinin-neuraminidase from parainfluenza virus, and host stress proteins caused by viral infections. After activation by the activating-receptors and localization to the site of infection, NK cells protect our body by three main mechanisms: Cytokine secretion, release of cytolytic granules, and death receptor-mediated cytolysis [20]. NK cells secrete several cytokines such as interferon-γ, tumor necrosis factor-α, granulocyte macrophage colony-stimulating factor (GM-CSF), and chemokines such as MIP-1a, MIP-1b, interleukin 8 (IL-8), and RANTES that can modulate the function of the innate and adaptive immune systems [21,22,23]. NK cells secrete perforins and granzymes, a family of serine proteases, after the formation of immunological synapses [24,25,26]. Perforin makes holes in the cell membrane and granzymes disrupt the nucleus after cell entry. NK cells can also induce apoptosis of target cells by expressing ligands that can activate death receptors on the target cells [27].

Because an unlimited number of NK cells can be derived from human pluripotent stem cells for off-the-shelf immune cell therapy, several groups have worked on generating NK cells from human pluripotent stem cells. The published methods can be categorized depending on their use of xenogeneic stromal cell lines and serum. Early protocols utilized mouse stromal cells (S17 or M210) for hematopoietic differentiation, and the differentiated cells were sorted and plated onto EL08-1D2 stromal cells with cytokines such as IL-15, IL-7, IL-3, and FLT3L, generating CD56^+^ CD45^+^ NK cells [28,29,30]. The differentiated NK cells expressed inhibitory and activating receptors such as CD16 and killer cell Ig-like receptors. In addition, the differentiated NK cells could lyse human tumor cells by both direct cell-mediated cytotoxicity and antibody-dependent cellular cytotoxicity. Considering potential clinical applications of human pluripotent stem cell-derived NK cells, a differentiation protocol that does not use xenogeneic cells and serum is essential. A serum-dependent and partial xenogeneic cell-dependent protocol has been reported [31]. In the protocol, hematopoietic progenitor cells differentiated by the spin embryoid body (EB) method were plated on EL08-1D2 stromal cells for 28–35 days with cytokines IL-15, IL-7, IL-3, and FLT3. Compared to xenogeneic cell- and serum-dependent protocols, the partial xenogeneic cell-dependent protocol resulted in comparable levels of C56^+^CD45^+^ NK cell differentiation and activity. A xenogeneic cell- and serum-free protocol was developed to generate more clinically relevant NK cells [32,33]. The protocol generated CD34^+^CD43^+^ hematopoietic progenitor cells by the spin EB method and the cells were further differentiated using membrane-bound IL-21-expressing artificial antigen-presenting cells.

### 2.2. Pluripotent Stem Cell-Derived-Macrophages

Like NK cells, macrophages are essential innate immune cells. These cells play critical roles in inflammation and in the protection of our body from pathogens such as bacteria and tumor cells. Different types of macrophages originate from different sites that include the yolk sac, the fetal liver, and the bone marrow. Yolk sac-derived macrophages reside in the brain as microglia [34]. Further, yolk sac-derived macrophages populate the fetal liver to generate the majority of tissue-resident macrophages (TRMs). The TRMs derived from the yolk sac and the fetal liver self-renew throughout adult life [35]. Bone marrow-derived macrophages have a relatively short life span and colonize tissues only after inflammation [36]. TRMs have diverse functions in different tissues and are important for homeostatic host protection and the response to tissue injury. In the brain, CD95L produced by TRMs (microglia) regulates neurovascular development [37]. In the bone, TRMs are involved in bone resorption [38]. In the heart, macrophages facilitate electrical conduction [39]. As an immunotherapeutic cell, macrophages use pattern recognition receptors (PRRs) to detect invaders such as bacteria. PRRs are categorized into four different classes that include Toll-like receptors and C-type lectin receptors, as well as cytoplasmic proteins such as retinoic acid-inducible gene (RIG)-I-like receptors and NOD-like receptors [40]. After PRRs bind to bacterial ligands, intracellular signaling pathways trigger actin polymerization and the formation of the phagocytic cup [41]. After phagocytosis of the bacteria, macrophages move into tissues and ultimately to lymph nodes to present digested antigens to T cells, triggering a T cell immune response.

Several protocols for macrophage differentiation from human pluripotent stem cells have been reported. A serum-free and xenogeneic cell-dependent protocol has been developed [42]. The authors differentiated human iPS cells grown on a mouse feeder layer into EBs; these cells were further differentiated into macrophages using IL-3, M-CSF, G-CSF, and GM-CSF. A xenogeneic cell- and serum-free differentiation protocol, in which cells in spin EBs were induced to mature into macrophages, has also been reported [43]. The macrophages derived from human pluripotent stem cells exhibit authentic macrophage functions. For example, these differentiated cells showed phagocytosis of opsonized yeast, endocytosis of lipoprotein, differentiation by INF-γ and IL-4, and secretion of cytokines in response to lipopolysaccharide [43]. Interestingly, human pluripotent stem cell-derived macrophages differentiated into specialized microglia-like cells when they were co-cultured with neurons in vitro. Moreover, in vivo, the differentiated macrophages were further differentiated into microglia and alveolar macrophages after injection into the brain and lung, respectively [44]. These results suggest that macrophages differentiated from human pluripotent stem cells in vitro share characteristics with macrophages that develop naturally in the body.

### 2.3. Pluripotent Stem Cell-Derived T Cells

T cells form the keystone of our body’s immune system, playing a critical role in carrying out cell-mediated immune responses (adaptive immune system). T cells protect our body by detecting foreign molecules expressed on the surface of antigen presenting cells with the help of the major histocompatibility complex (MHC). CD4+ T cells (helper T cells) help other immune cells by regulating T cell cytokine secretion. The cytokines released by CD4+ T cells activate or inactivate CD8+ cytotoxic T cells and macrophages. In addition, cytokines can affect antibody class switching by B cells [45]. CD8+ cytotoxic T cells detect antigens presented by class I MHCs and common antigens produced by cancer cells or viruses. CD8+ cytotoxic T cells take advantage of their T cell receptors (TCRs) to bind to foreign antigens. The TCR complex is composed of six polypeptide subunits. In most CD8+ cytotoxic T cells, the αβ subunits of the TCR function as immunoglobulin-like variable domains that recognize peptide antigens associated with class I MHCs expressed by antigen-presenting cells. TCRαβ subunits complex with CD3 subunits γ, δ, ε, and ζ, which are required for T cell signal transduction [46]. Upon binding to foreign peptides, CD8+ cytotoxic T cells secrete molecules such as perforin, granzymes, and granulysin. The cooperative action of these molecules induces apoptosis of target cells. In addition to this mechanism, activated CD8+ cytotoxic T cells express the FAS ligand, which allows CD8+ cytotoxic T cells to bind to FAS-expressing cells to induce apoptosis [47].

Several reports have presented differentiation protocols that enable the generation of functional T cells from human pluripotent stem cells. When used as a stromal cell line, the mouse bone marrow-derived OP9 cell line can differentiate pluripotent stem cells to CD34+ hematopoietic cells. Further, information gained from the in vitro differentiation of hematopoietic progenitor cells from bone marrow, peripheral blood, and cord blood into T cells indicated that Notch signaling is required for T cell derivation. Therefore, OP9 cells were engineered to express the Notch ligand, Delta-like ligand 1 (Dll-1), and termed OP9-DL1. Using the OP9-DL1 cell line, human pluripotent stem cells were differentiated into T cells [48,49]. The protocol generated functionally mature CD4+ and CD8+ TCR αβ T cells. Recently, T cell differentiation using DLL-4 instead of Dll-1 was reported. Montel-Hagen et al., showed conventional T cell differentiation from human pluripotent stem cells using organoid [50]. They sequentially differentiated the pluripotent stem cells from mesoderm through hematopoietic specification to conventional CD3^+^CD8αβ^+^ and CD3^+^CD4^+^ T cells by organoid formation mixed with human DLL4-expressing MS5 cells. Kumar et al., demonstrated hematovascular mesodermal progenitor population with KDR^hi^CD31^-^ phenotype produces T cells when cultured on OP9-DLL4 [51]. Notch activation was required for efficient T cell generation with high proliferation potential at the hematovascular mesoderm stage. Recently, one interesting paper described the rejuvenation of exhausted T cells [52]. Blood cells from a patient chronically infected with HIV-1 were collected, and T cells were reprogrammed with iPS reprogramming factors expressed from retroviruses. The iPS cells were re-differentiated into T cells using the OP9-DL1 cell line. The differentiated T cells showed high proliferative activity and elongated telomeres, suggesting that exhausted T cells from chronically infected patients can be rejuvenated during iPS reprogramming.

## 3. Chimeric Antigen Receptor

**Figure 2:**
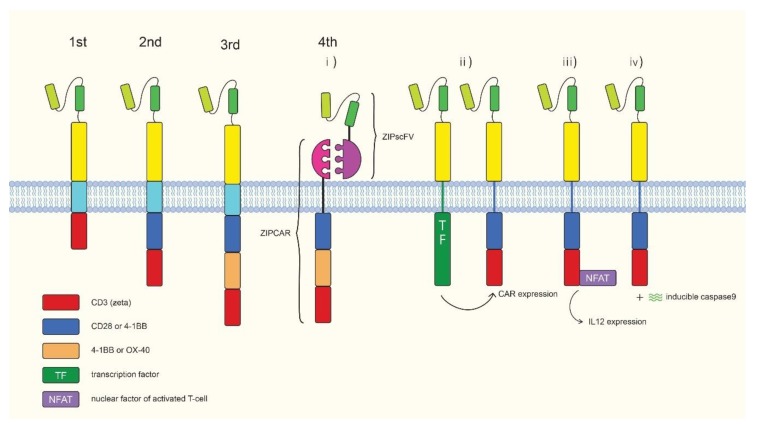
Overview of chimeric antigen receptors. Frist generation (chimeric antigen receptors) CARs contain the single chain variable (scFv) domain from a monoclonal antibody, and an intracellular signaling domain of CD3 zeta. Second generation CAR adds a co-stimulatory domain (CD28 or 4-1BB) on the first generation CAR. Third generation CAR contain two co-stimulatory domains (CD28, 4-1BB or OX40). Fourth generation CARs. The newest version is equipped with the features such as flexibility against antigens, or controllable on/off switch to increase safety and versatility of the treatment. (i) split, universal, and programmable (SUPRA) CAR-T that has a flexible antigen detection activity. (ii) Synnotch CAR-T. After binding of the first antigen, the intracellular transcription factor (TF) domain is cleaved and translocated into the nucleus to induce second CAR expression. (iii) T cell redirected universal cytokine killing (TRUCK) CAR-T cells. Once the first antigen is detected, the nuclear factor of activated T cell (NFAT) induces IL12 expression. (iv) Induced caspase 9 (iC9) CAR-T. CAR-T cells express inducible caspase 9 by chemical inducer of dimerization. This system efficiently eliminates 85%~90% of CAR-T cells improving the safety of CAR-T cells.

Chimeric antigen receptors (CARs) are synthetic receptors. The basic CAR structure consists of a tumor-specific antigen detection domain derived from a monoclonal antibody, a T cell activation domain usually derived from the CD3 ζ chain, and a linker derived from the T cell transmembrane domain that bridges the two domains (Figure 2). First generation CARs were only composed of these three structures, with subsequent generations having additional structures. When CAR is expressed in T cells (CAR-T cells), the antigen detection domain of the CAR guides the engineered T cell to tumor cells. Upon binding to tumor-specific antigens, the T cell activation domain induces downstream signaling to activate the T cell. The first generation CAR is only composed of these three structures, with subsequent generations having additional structures. When CAR is expressed in T cells (CAR-T cells), the CAR guides the engineered T cell to the tumor cells using its antigen detection domain. Upon binding to tumor specific antigens, the T cell activation domain relays downstream signaling to activate the T cell. The first CAR-T cell concept was developed by Dr. Zelig Eshhar [53]. In this study, they generated a double chain chimeric receptor composed of the V region domains of the heavy (VH) and the light (VL) chains of an anti-2,4,6-trinitrophenyl (TNP) antibody fused to the T cell receptor constant (C) domains. The T-cells transfected with this chimeric T cell receptor specifically killed TNP-bearing target cells and produced IL-2. The T-cells showed a response to immobilized TNP-protein conjugates, and cellular processing and presentation of antigens by major histocompatibility complex (MHC) were not necessary. First generation CAR-T cells of the present form were developed by Zelig Eshhar et al. [54]. This CAR was composed of a single-chain Fv domain (scFv) from an anti-TNP antibody and CD3 γ or ζ chains. However, these first generation CAR-T cells did not exert satisfactory effects and showed limited proliferation and short survival times. To enhance CAR-T activity, a second generation CAR was developed [55]. A co-stimulatory domain such as CD28 or 4-1BB was built into T cells for full activation. The two FDA-approved CAR-T products are second generation CARs [56,57]. Third generation CARs utilize two co-stimulatory domains together to maximize T cell activation [58,59,60,61]. Fourth generation CARs contain additional functional domains such as a suicide gene and a controllable on-off switch, enhancing T cell activity [62]. A controllable on-off switch was introduced into CAR-T cells by using human caspase-9 fused to the human FK506 binding protein. Using chemical inducer of dimerization results in apoptosis of cells expressing the fusion protein. The system efficiently eliminated 85%~90% of CAR-T cells, improving the safety of CAR-T cells [63]. Roybal et al. published about a dual CAR system in which one synthetic Notch receptor detected an antigen leading to inducible expression of a second CAR that recognized another antigen [64]. This dual CAR system allows the CAR-T cells to decide when to activate in a more precise way. Another interesting CAR is split, universal, and programmable (SUPRA) CAR. The SUPRA CAR system is composed of a zipCAR and a zipscFv. A zipCAR has a leucine zipper fused to a T cell activation domain as the extracellular portion of the CAR. A zipscFv has an antigen detection scFv fused to a cognate leucine zipper that can bind to the leucine zipper on the zipCAR. By allowing for changes in zipCAR function, the SUPRA CAR system endows T cells with target antigen flexibility and confers the ability to fine tune T-cell activation [65]. CAR-T cells have shown remarkable clinical outcomes [66,67,68,69]. Based on the clinical outcomes of CAR-T cells in hematological malignancies, two CAR-T products, Kymriah and Yescarta, were approved by the FDA in 2017 [5]. These approved CAR-T products utilize CD19 CARs that detects CD19 expression on B cells. Since then, many clinical trials have been registered to enhance efficacy and safety of CAR-T cell therapy using gene editing technologies and fourth generation CAR-T cells (Table 1, Clinicaltrials.gov). With the unprecedented success of CAR-T cell therapy in blood cancers, researchers became enthusiastic to apply the fruits of this successes to solid tumors. Unfortunately, when CAR-T cells were applied to solid tumors, the outcomes were less successful than they were for hematological malignancies [70]. Possible reasons for these disappointing results could include the following factors. First, solid tumors protect themselves by forming a massive tissue with physical barriers that are difficult for CAR-T cells to penetrate [71]. Second, the solid tumor microenvironment hinders CAR-T antitumor activity by secreting inhibitory molecules or providing resident inhibitory cells [72]. Lastly, tumor-specific antigens in solid tumors are heterogeneous, thereby limiting the effectiveness of monoclonal antibody-guided therapy [73]. In addition to these hurdles, maintaining the quality of patient-derived T cells is very challenging. In autologous CAR-T cell therapy, a patient’s condition strongly influences the quality of T cells; variation in T cell quality impacts the efficiency of manufacturing and the treatment potency of the therapy [74]. In addition, the temporal and spatial limitations of manufacturing could hamper broad clinical applications of CAR-T cell therapy. Currently, cell production for autologous CAR-T cell therapy requires around 14~21 days and occurs at multiple sites [75]. These features of CAR-T cell manufacturing inevitably make the end product expensive. CD19 CAR-T cell therapy costs $350,000–$500,000. These limitations warrant the development of a new method to harness CAR-T cell technology to ensure CAR-T cell therapy fully satisfies its medical potential.

## 4. Stem Cell-Derived CAR Immune Cells

Use of gene editing technology, such as the clustered regularly interspaced short palindromic repeats (CRISPR)/Cas9 system, during the generation of human pluripotent stem cell-derived immune cells could mitigate current unmet medical needs associated with the use of immune cell therapy in the clinic. Unmet medical needs related to quality control, manufacturing, and price are due to autologous transplantation, which could be resolved by generating “off-the-shelf” immune cells. For such cells to be useful, the genes related to immune responses must be modified by gene editing. For example, gene editing tools such as zinc finger nucleases, transcription activator-like effector nucleases (TALENs), and CRISPR could be utilized to modify genes encoding products such as human leukocyte antigen (HLA). However, it is well known that primary immune cells are refractory to transfection and fragile afterwards. Because primary immune cells are hostile to gene editing, human pluripotent stem cells, which can easily be engineered by virus-, RNA-, plasmid-, or protein-mediated transfection, could be an ideal cell source for generating “off-the-shelf” cells. Moreover, human pluripotent stem cells can be clonally selected after modification due to their indefinite proliferation capacity. Therefore, human pluripotent stem cells could be engineered into a universally compatible cell line to supply unlimited “off-the-shelf” immunotherapeutic cells. Two recent papers showed the possibility of generating hypoimmunogenic iPS cells that evade immune rejection by host cells after transplantation [76,77]. HLA class I is a key molecule that causes immune rejection of transplanted cells in allogeneic recipients. Although transplanted cells lacking HLA class I can evade CD8+ cell attack, these cells can still be surveilled by NK cells, leading to lysis, in the ‘missing self’ response. To prevent NK cell-mediated cell lysis, the authors overexpressed HLA-E, which is minimally polymorphic. They knocked-in the *HLA-E* gene into the *beta-2 Microglobulin* (*B2M*) locus to disrupt HLA class I expression. The HLA class I negative, HLA-E expressing iPS cells were differentiated into CD45+ hematopoietic cells and transplanted into allogeneic recipient mice [76]. The cell modifications prevented NK cell-mediated lysis. The other paper showed that HLA class I, II knockout, together with overexpression of CD47, a “don’t eat me” signal, can generate hypoimmunogenic iPS cells. The engineered human iPS cells were differentiated into endothelial cells, smooth muscle cells, and cardiomyocytes, and transplanted into fully MHC-mismatched allogeneic recipients. Interestingly, these cells evaded immune rejection [77]. To prevent graft versus host disease, the genes encoding the TCR α and β subunits should be knocked out by different gene editing tools [78].

The Sadelain group showed that CAR-T cells can be generated from human iPS cells [79]. They used lentiviral transduction into iPS cells to express a second generation CAR that detects CD19. The CAR-expressing iPS cells were successfully differentiated into T cells using the OP9-DL1 cell line, suggesting that CAR-T generation from human pluripotent stem cells is feasible. Because the CRISPR/Cas9 system enables multiplex gene editing, and CAR-T cells were generated from iPS cells, CAR-T cells derived from human pluripotent stem cells with +HLA-/TCR-/HLA-E or CD47 overexpression properties could easily be produced from human pluripotent stem cells. Positive clones could be selected, and the selected cells could be differentiated into “off-the-shelf” immunotherapeutic cells.

NK cells do not require HLA matching to exert their activity, and therefore they can function as “off-the-shelf” cells without any modifications [80]. Li et al. showed CAR-NK generation from human iPS cells [81]. Human iPS cells were transfected with a plasmid that encodes scFv specific for human mesothelin, the 2B4 co-stimulatory domain, and CD3 ζ. Human iPS-derived CAR-NK cells showed in vivo cytotoxic activity toward tumor cells comparable to CAR-T cells, but with less overall toxicity [81]. These results indicate that CAR-NK cells derived from human pluripotent stem cells could be a valuable method for generating “off-the-shelf” allogeneic therapeutics. Ten clinical trials of CAR-NK have been registered (Table 2, Clinicaltrials.gov). It is notable that fourth generation CAR is being under examination for CAR-NK. In solid tumors, the tumor microenvironment prevents entry of CAR-T cells. Interestingly, macrophages have a unique ability to penetrating solid tumors. Based on this observation, one group tested CAR incorporation into a mouse macrophage cell line [82]. They showed that CAR expression increased the phagocytic activity of macrophages, suggesting that it is possible to develop human pluripotent stem cell-derived CAR-macrophages and giving hope to the idea of developing a new immune therapy for solid tumors.

## 5. Conclusions

**Figure 3:**
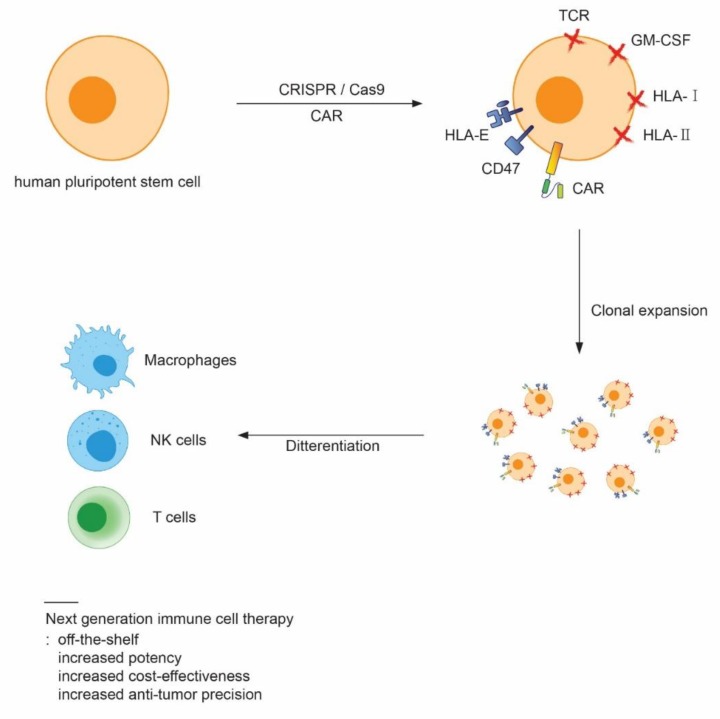
Overview of future strategies for developing next generation immune cell therapy from human pluripotent stem cells. By deploying CAR and clustered regularly interspaced short palindromic repeats (CRISPR/Cas9 technologies, human pluripotent stem cells can be engineered to knockout the genes related to immune rejection, cytokine release syndrome, or to express fourth generation CAR, which can be utilized for precise anti-tumor activity and safety. The engineered human pluripotent stem cells can be clonally selected and expanded. The clonally expanded cells differentiated to immune cells for the therapy. This approach can generate next generation immune cells, which can pave a new way for developing immune cell therapy with off-the shelf, increased potency, increased cost-effectiveness, and an increase in anti-tumor activity precision.

Immune cell therapy is a transformative treatment for cancers that are not curable by conventional treatments [2,3]. Enormous effort is being applied to develop immune cell therapies [4]. In addition to merely developing immune cells for therapy, making the immune cells safer, more potent, and more cost-effective, is critical for their clinical application. To this end, engineering immune cells with CRISPR and CAR technologies is being investigated. CRISPR/Cas9 tools provide a simple means of generating multiplex gene modifications, and to date 11 clinical trials for gene-edited CAR-T cells have been registered (clinicaltrial.gov) [83]. CAR engineering enables immune cells to detect tumor-specific antigens and to be activated upon binding to their cognate antigens. Currently, 10 clinical trials for CAR-NK (Table 2, clinicaltrial.gov) and 220 trials for CAR-T are registered [84]. The number of CAR technology-related clinical trials is increasing exponentially, indicating a growing enthusiasm for immune cell therapy [84]. Amidst this enthusiasm, the biggest challenge from a technical perspective is that primary immune cells are difficult to engineer. The cells are refractory to exogenous gene expression and fragile after the engineering process. In addition, primary cells have an intrinsically limited ability to proliferate and, therefore, it is difficult to perform clonal selection and to obtain enough clonally-selected cells for clinical applications. The generation of immune cells from human pluripotent stem cells has been on the cusp of regenerative medicine because these cells can easily be engineered, and pluripotent stem cell-derived immune cells can be utilized in cell therapy. Moreover, with their ability to proliferate indefinitely, human pluripotent stem cells could be an ideal alternative for providing unlimited amounts of improved immune cells through engineering and clonal selection [6]. For example, one of the most challenging adverse effects remaining in CAR-T cell therapy is cytokine release syndrome (CRS) [50]. Characteristic CRS symptoms are high fever, hypotension, hypoxia, and respiratory distress. In some cases, organ dysfunction and life-threatening complications have been caused by CRS. A recent paper showed that TALEN-mediated genetic ablation of the gene encoding GM-CSF in CAR-T cells can prevent CRS [85]. These results could be applied to human pluripotent stem cells to generate cells that were less prone to causing CRS using CRISPR/Cas9, after which the engineered cells could be clonally selected and differentiated into CAR-T cells. Therefore, through the deployment of CRISPR/Cas9 technology and fourth generation CARs, human pluripotent stem cells could enhance next generation immune cell therapy in terms of safety, cost, and activity. However, the generation of CAR-immune cells from human pluripotent stem cells is at an early stage. A limited number of papers have reported the generation of CAR-T and CAR-NK cells from human iPS cells [79,81]. No paper has yet reported successful CAR-macrophage generation from human pluripotent stem cells. Therefore, robust protocols for producing CAR-immune cells from human pluripotent stem cells must be developed to achieve next generation immune cell therapy. In conclusion, CRISPR-modified human pluripotent stem cell-derived CAR-immune cells hold an unprecedented potential for treating previously incurable cancers. The resulting next generation immune cell therapy may solve limitations of current therapies by achieving improvements such as off-the-shelf availability, increased potency, increased cost-effectiveness, and increased precision of anti-tumor activity (Figure 3).

## Figures and Tables

**Table 1:** Clinical trials of gene edited- or fourth generation CAR-T cell therapy.

NCT Number	T-Cell	Indication	Country	Group	Features
NCT03399448	NYESO-1 TCR-T	MM/Melanoma/Synovial Sarcoma/Myeloid/Round cell iposarcoma	US	U. Penn.	TCR/PD-1 KO
NCT03166878	CD19 CAR-T	B-cell Leukemia/Lymphoma	China	Chinese PLA General Hospital	TCR/B2M KO
NCT03398967	CD19 AND CD20/22 CAR-T	B-cell Leukemia/Lymphoma	China	Chinese PLA General Hospital	TCR KO
NCT03081715	Primary T cell	Esophageal Cancer	China	Hangzhou Cancer Hospital Anhui Kedgene Biotechnology	PD-1 KO
NCT02863913		Invasive Bladder Cancer Stage IV	China	Peking Univ./Cell Biotech	
NCT02867345		Hormone Refractory Prostate Cancer	China	Peking Univ./Cell Biotech	
NCT02867332		Metastatic Renal Cell Carcinoma	China	Peking Univ./Cell Biotech	
NCT02793856		Metastatic Non-small Cell Lung Cancer	China	Sichuan Univ./Chengdu MedGenCell	
NCT03044743	EBV-CTL	Gastric Carcinoma/Nasopharyngeal Carcinoma/T-cell Lymphoma/Adult Hodgkin Lymphoma/Diffuse Large B-cell Lymphoma	China	Nanjing Drum Tower Hospital	
NCT02746952	CD19 CAR-T	Relapsed/refractory B-All	US/Europe	Servier	TCR/CD52 KO
NCT03190278	CD123 CAR-T	Acute Myeloid Leukemia	US	Cellectis	TCR KO
NCT03050190	CD19 CAR-T	Relapsed and Refractory B cell Malignancies	China	Shenzhen Geno-Immune Medical Institute	Inducible apoptotic caspase 9
NCT02247609		High-risk and Refractory B cell Lymphomas		Peking Univ.	
NCT02968472		Relapsed and Refractory B cell Leukemia		The First People’s Hospital of Yunnan	
NCT03098355	CD19 and CD22 CAR-T	Refractory and/or Recurrent B cell Malignancies		Zhujiang Hospital	
NCT02274584	CD30 CAR-T	CD 30 Positive Lymphomas		Peking Univ.	
NCT03185468	PSMA, Fra CAR-T	Bladder Cancer		Shenzhen Gene-Immune Medical Institute	

Fourth generation CAR-T with inducible on/off switch by caspase 9 to enhance safety is under clinical trials. Using CRISPR/Cas9 and transcription activator-like effector nucleases (TALEN), T-cell receptor or PD-1 were deleted (knockout, KO) for generating off-the-shelf CAR-T cells or overcoming immune check point, respectively.

**Table 2:** Clinical trials of CAR-NK cell therapy. Ten trials are ongoing, and two trials are evaluating fourth generation CAR which expresses inducible apoptotic caspase 9, or inducible apoptotic caspase 9 with IL15.

NCT Number	NK-Cell	Indication	Country	Group	Features
NCT03692767	CD22 CAR-NK	Relapsed and Refractory B cell Leukemia	China	Allife Medical Science and Technology Co., Ltd	-
NCT03690310	CD19 CAR-NK	Relapsed and Refractory B cell Leukemia	China		-
NCT03398967	Mesothelin CAR-NK	Epithelial Ovarian Cancer	China		-
NCT03692663	PSMA CAR-NK	Castration-Resistant Prostate Cancer	China		-
NCT03824964	CD19/CD22 CAR-NK	Relapsed and Refractory B cell Leukemia	China		-
NCT03415100	NKG2D-CAR-NK	Metastatic Solid Tumours	China	The Third Affiliated Hospital of Guangzhou Medical Univ.	-
NCT02944162	CD33 CAR-NK	Relapsed/Refractory CD33+AML	China	PersonGen BioTherapeutics Co., Ltd	-
NCT02892695	CD19 CAR-NK	CD19 Positive Leukemia and Lymphoma	China		-
NCT03579927	CD19 CAR-NK	B-cell Lymphoma	US	M.D. Anderson Cancer Center	Inducible apoptotic caspase 9
NCT03056339	CD19 CAR-NK	B Lymphoid Malignancies	US		Inducible apoptotic caspase 9 + IL15
